# Modeling the effect of levothyroxine therapy on bone mass density in postmenopausal women: a different approach leads to new inference

**DOI:** 10.1186/1742-4682-4-23

**Published:** 2007-06-09

**Authors:** Babak Mohammadi, Vahid Haghpanah, Seyed Mohammad Tavangar, Bagher Larijani

**Affiliations:** 1 Endocrinology and Metabolism Research Center (EMRC), Tehran University of Medical Sciences, Tehran, Iran

## Abstract

**Background:**

The diagnosis, treatment and prevention of osteoporosis is a national health emergency. Osteoporosis quietly progresses without symptoms until late stage complications occur. Older patients are more commonly at risk of fractures due to osteoporosis. The fracture risk increases when suppressive doses of levothyroxine are administered especially in postmenopausal women. The question is; "When should bone mass density be tested in postmenopausal women after the initiation of suppressive levothyroxine therapy?". Standard guidelines for the prevention of osteoporosis suggest that follow-up be done in 1 to 2 years. We were interested in predicting the level of bone mass density in postmenopausal women after the initiation of suppressive levothyroxine therapy with a novel approach.

**Methods:**

The study used data from the literature on the influence of exogenous thyroid hormones on bone mass density. Four cubic polynomial equations were obtained by curve fitting for Ward's triangle, trochanter, spine and femoral neck. The behaviors of the models were investigated by statistical and mathematical analyses.

**Results:**

There are four points of inflexion on the graphs of the first derivatives of the equations with respect to time at about 6, 5, 7 and 5 months. In other words, there is a maximum speed of bone loss around the 6^th ^month after the start of suppressive L-thyroxine therapy in post-menopausal women.

**Conclusion:**

It seems reasonable to check bone mass density at the 6^th ^month of therapy. More research is needed to explain the cause and to confirm the clinical application of this phenomenon for osteoporosis, but such an approach can be used as a guide to future experimentation. The investigation of change over time may lead to more sophisticated decision making in a wide variety of clinical problems.

## Background

### Osteoporosis

The World Health Organization (WHO) defines osteoporosis as bone mineral density more than or equal to -2.5 Standard Deviation (SD) below the young adult mean [1]. This definition is the one most often used by radiologists when they measure bone density and it gives the physician an idea of fracture risk. Non-modifiable risk factors include: female gender, Caucasian or Asian race, family history [2] and a personal history of fracture as an adult. Modifiable factors include: smoking, inadequate dietary calcium, estrogen deficiency [3], excess dietary sodium, alcoholism, low body weight (<57.6 kg), inactivity and lack of weight bearing exercise [4]. Secondary causes of osteoporosis include a broad range of diseases and medications. Drugs may include corticosteroids, anticonvulsants, heparin, aluminum and thyroxine. Secondary osteoporosis may be due to hyperparathyroidism, hyperthyroidism, diabetes, chronic renal failure, scoliosis, gonadal insufficiency, multiple myeloma, lymphoma, chronic obstructive pulmonary disease, rheumatoid arthritis, sarcoidosis, and malabsorption syndromes among several other conditions.

### The effect of thyroid hormone on bone turnover

Thyrotoxicosis increases bone turnover in favor of net bone resorption [5]. Thyroid disease and osteoporosis are common problems often managed by primary care physicians. Thyroid hormone preparations are widely used either at replacement doses to correct hypothyroidism of any etiology (except transient hypothyroidism during the recovery phase of subacute thyroiditis) and for simple (nonendemic) goiter and chronic lymphocytic (Hashimoto's) thyroiditis; or at suppressive doses to supress thyrotropin (thyroid-stimulating hormone) secretion in patients with differentiated thyroid carcinoma after total thyroidectomy or with diffuse nodular nontoxic goiter. In order to suppress thyrotropin secretion, it is necessary to administer slightly supraphysiological doses of thyroxine and reduced bone density and bone mass is a possible adverse effect of this therapy [6]. The availability of sensitive thyrotropin assays allows effective biochemical monitoring of both replacement and suppressive therapy to be conducted [7].

Frank hyperthyroidism is a recognized risk factor for osteoporosis, but the effects of subclinical hyperthyroidism on bone mass density are less well defined [8]. In two cross-sectional studies of patients with subclinical hyperthyroidism due to multinodular goiter, there were statistically and clinically significantly lower bone mineral densities at the femoral neck and radius than in age-matched controls [9,10]. Some data confirm that postmenopausal women receiving suppressive doses of T_4 _for thyroid carcinoma have diminished bone mineral measurements and are at risk of osteoporosis [11-13]. Estrogen use also appears to negate thyroid hormone-associated loss of bone density in postmenopausal women [14]. Also there is a risk of bone loss in post-menopausal females with a previous history of thyrotoxicosis treated with radioiodine [15]. It has been shown that women on long-term TSH-suppressive doses of L-T_4 _have reduced bone mass density (BMD) at various skeletal sites, which may increase fracture risks. It has therefore been recommended that TSH-suppressive doses of thyroid hormone should only be prescribed when appropriate and for no longer than necessary to minimize this adverse effect of excessive doses on bone [16]. A prospective study of bone loss in pre- and post-menopausal women on L-thyroxine therapy for non-toxic goiter suggests that TSH-suppressive therapy with L-thyroxine for non-toxic goiter significantly increases the bone mineral turnover and might contribute to a reduction of BMD, more marked in cortical bone, in both pre- and post-menopausal women [17].

On the other hand, some studies have suggested that thyroxine therapy alone is not a major risk factor for the development of osteoporosis [18-26] and bone mass reduction could be transient and reversible because new bone is formed at the end of the resorptive sequence [27]. Some data have shown a small detrimental effect of cautious L-T_4 _suppressive therapy on bone mass assessed by dual energy x-ray absorptiometry (DEXA) [28]. Despite many studies, confusion still exists about the effect of thyroid hormone on skeletal health [29].

Data selected from cross-sectional studies, longitudinal studies, and meta-analyses with appropriate control groups (patients matched for age, sex, and menopausal status) were reviewed in comparison with established databases or thyroid state defined by TSH level or thyroid hormone dose. Overall, hyperthyroidism and use of thyroid hormone to suppress TSH because of thyroid cancer, goiters, or nodules seemed to have an adverse effect on bone, especially in postmenopausal women; the largest effect was on cortical bone. Thyroid hormone replacement seemed to have a minimal clinical effect on bone. The study suggested that women with a history of hyperthyroidism or TSH suppression by thyroid hormone should have skeletal status assessed by bone mineral densitometry, preferably at a site containing cortical bone, such as the hip or forearm [30].

## Subjects and methods

### Pre-assumptions

There are many factors influencing the behavior of our system, which cannot be captured in a usable model. So the first task is to simplify the model by reducing the number of factors under consideration. The independent variables may include; time (the duration of therapy), gender, race, family and personal medical history, diet, hormonal changes, medications, alcohol ingestion, physical activity, medical conditions and diseases, etc. The dependent variable is BMD. To simplify the problem we assume that the patients are postmenopausal women receiving suppressive LT4 therapy. It is desired to find the value of BMD as a function of the duration and the dose of LT4 therapy. Any remaining factor can be regarded as a special case of a variable with unchanging numerical values, or in other word as a constant.

### Topological considerations

The numerical value of bone mass density is determined by measurement. The range of the variable may differ depending on the characters and methods of measurement and is the set of all points lying between the healthy and frankly osteoporotic situations. To each value of time *t *∈ *T*, and dose *m *∈ *M*, within certain ranges there corresponds one value of the variable, bone mass density *d *∈ *D*. Consider the sets *T*, *D *and *M*. The product set *T *× *D *× *M *is defined as *T *× *D *× *M *= {(*t*, *d*, *m*): *t *∈ *T*, *d *∈ *D*, *m *∈ *M*}. But the set *M *can be written as *M *= {*m *: *m *= suppressive dose *s*, *m *= replacement dose *r*}.

Symbolically, the relationship can be stated in function notation as BMD = *f *(time, dose), with two submodels, *d *= *f*_*s*_(*t*) for levothyroxine suppressive therapy, and *d *= *f*_*r*_(*t*) for levothyroxine replacement therapy. To each function f : T → D there corresponds a relation in T × D given by the graph of *f*, {(*t*, *f*(*t*)):*t *∈ *T*}. The domain of *f *is *T*, and its range *f *[*T*] = {*f*(*t*):*t *∈ *T*} is the quantity of *d*, from the normal to the extreme osteoporotic state. As mentioned above we follow the case *d *= *f*_*s*_(*t*).

### Statistical analysis and investigating the behavior of the model

The independent variables time *t *and dose *m *are not random but are quantities preselected by the investigator and have no distributional properties. BMD *d *is also the response to time and dose. So the problem is to find a polynomial function *f *that would represent the relationship between *d *and *t*. The values of *t *at which turning points occur can be found by solving *f*'(*t*) = 0, where *f*'(*t*) is the first derivative of the function with respect to time. The corresponding values of *d *are then determined by substituting the *t *values found in *d *= *f*(*t*). Also, the type of each turning point can be tested via evaluating *f*"(*t*), the second derivative of the function with respect to time.

### Patients

Kung and Yeung [13] prospectively studied 46 postmenopausal Chinese women, aged 63.4 ± 7.0 yr, with carcinoma of thyroid after total thyroidectomy and radioactive iodine ablation for 2 years. The aim was to evaluate the rate of bone loss and to assess whether calcium supplementation with or without intranasal calcitonin was able to decrease the rate of bone loss. Among the patients, 34 were recruited randomly from the clinic to participate in a cross-sectional study and were shown to have decreased BMD. Two had suffered atraumatic fractures during T4 therapy. The other 12 were subsequently recruited if they satisfied the inclusion criteria. None of the patients had features of recurrent disease and did not require calcium replacement for hypoparathyroidism. All were receiving a stable dose of T4 for at least 1 yr in the form of levothyroxine sodium (L-T,) suppressive therapy, i.e. all patients had immeasurable TSH levels (<0.03 mIU/L). The subjects had had regular menstruation before the menopause and did not have late menarche, early menopause, or oophorectomy. None had received hormonal contraceptive agents in the past. All were nonsmokers and non-drinkers, and none was taking medications or drugs known to affect bone mineral metabolism. There was no known history of osteoporosis in the family.

**Figure 1:**
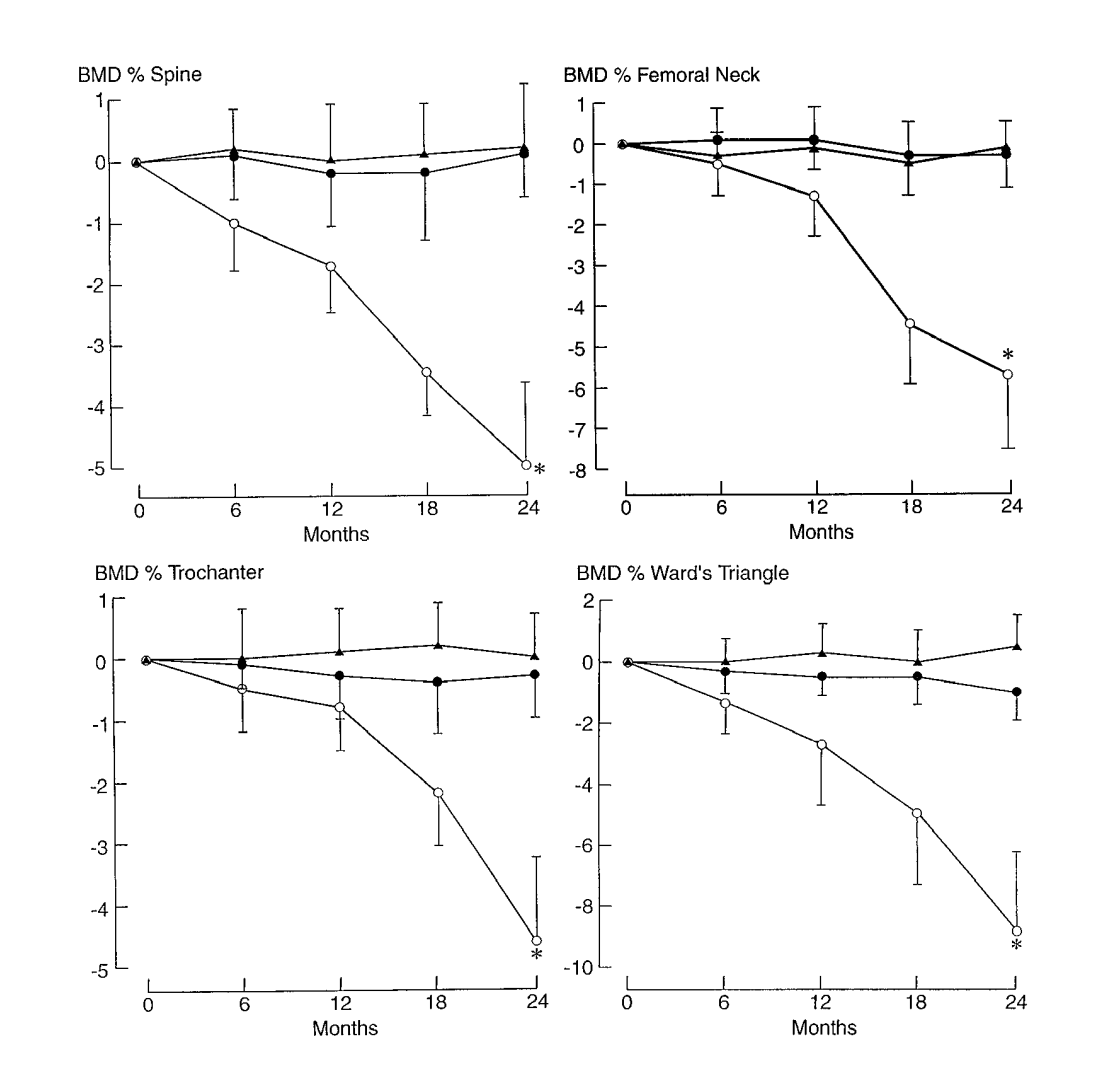
**Changes in regional BMD**. Changes in regional BMD in the spine, femoral neck, trochanter, and Ward's triangle in patients receiving T4-suppressive therapy treated with intranasal calcitonin plus calcium (▲), calcium alone (●), or placebo (○). Values are the mean ± SD. P < 0.05 vs. baseline reading. (With permission of Kung AW, Yeung SS. Prevention of bone loss induced by thyroxine suppressive therapy in postmenopausal women: the effect of calcium and calcitonin. J Clin Endocrinol Metab 1996; 81:1232-6)

All patients had received a stable dose of L-T4 for more than 1 yr. All had TSH levels of 0.03 mIU/L or less and an elevated free T4 (FT4) index, but normal T3 levels. The subjects were randomized into three groups: 1) intranasal calcitonin (200 IU daily) for 5 days/week plus 1000 mg calcium daily, 2) calcium alone, or 3) placebo. Total body and regional bone mineral density were measured by a dual energy x-ray absorptiometry bone densitometer at 6-month intervals. The results showed that both groups 1 and 2 had stable bone mass, whereas patients in group 3 showed significant bone loss at the end of 2 yr; there were no differences between groups 1 and 2. The authors concluded that T4-suppressive therapy is associated with bone loss in postmenopausal women, which could be prevented by either calcium supplementation or intranasal calcitonin (Figure 1)

## Results

**Figure 2:**
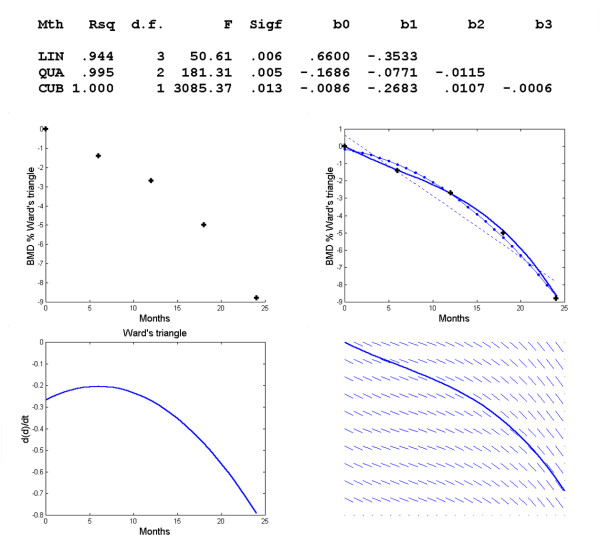
**Changes in BMD with respect to time in the Ward's triangle**. Polynomial regression for changes in regional BMD with respect to time in the Ward's triangle. The observed data (+) and the linear (--), quadratic (-.) and cubic (-) model, graph of *f*'(*t*) vs. *t *and direction field for the Ward's triangle.

We concentrated on mean changes in regional BMD at the spine, femoral neck, trochanter, and Ward's triangle in patients receiving T4-suppressive therapy treated with placebo. We estimated curve using the SPSS system for performing the regression for the response analysis. Initially regional bone mass density was examined in the Ward's triangle. The program fitted a cubic response model and also provided some results that are useful for determining the suitability of the model (Figure 2).

The coefficients in the last row yield the regression function *d *= -.0086-.2686*t*+.0107*t*^2^-.0006*t*^3^. The model was constructed graphically using MATLAB. Solving the equation *f*'(*t*) = 0 yields two complex roots, 5.9444 ± 10.6639i. We therefore solved *f*"(*t*) = 0 to find possible points of inflexion. The answer was *t *= 5.94444, or about 6 months, and this is compatible with the graph of *f*'(*t*) versus *t*. In the curve of BMD versus time, the slope is always negative. In the graph of *f*'(*t*) versus time, *f*'(*t*) = 0 reaches a non-zero maximum.

**Figure 3:**
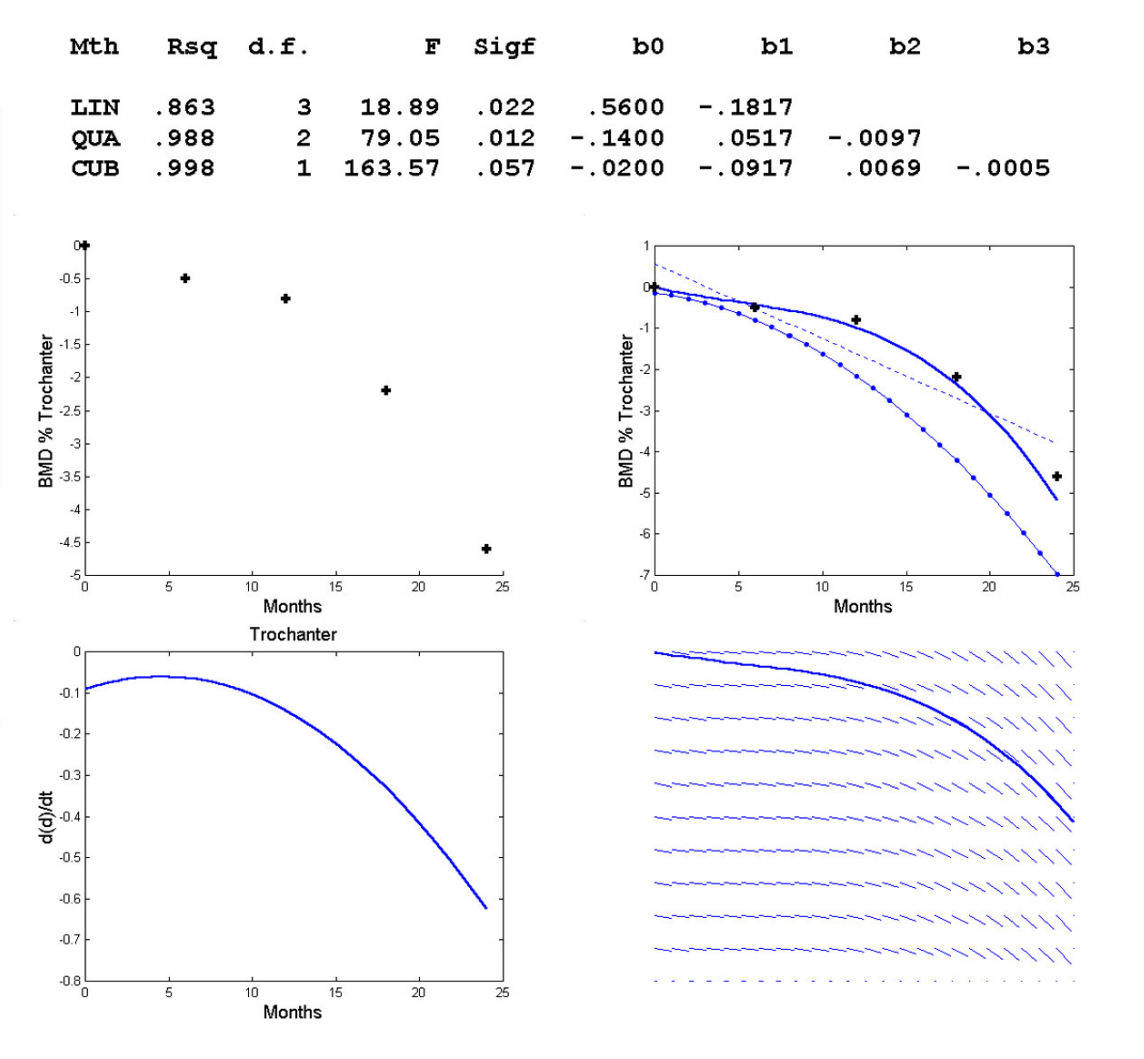
**Changes in BMD with respect to time in the trochanter**. Polynomial regression for changes in regional BMD with respect to time in the trochanter. The observed data (+) and the linear (--), quadratic (-.) and cubic (-) model, graph of *f*'(*t*) vs. *t *and direction field for the trochanter.

For the regional bone mass density in the trochanter the program again fitted a cubic response model (Figure 3). The regression function is *d *= -.0200-.0917*t*+.0069*t*^2^-.0005*t*^3^. Solving *f*"(*t*) = 0 yields *t *= 4.6 or about 5 months, at which there is a point of inflexion on the regression curve and *f*'(*t*) reaches a maximum value.

**Figure 4:**
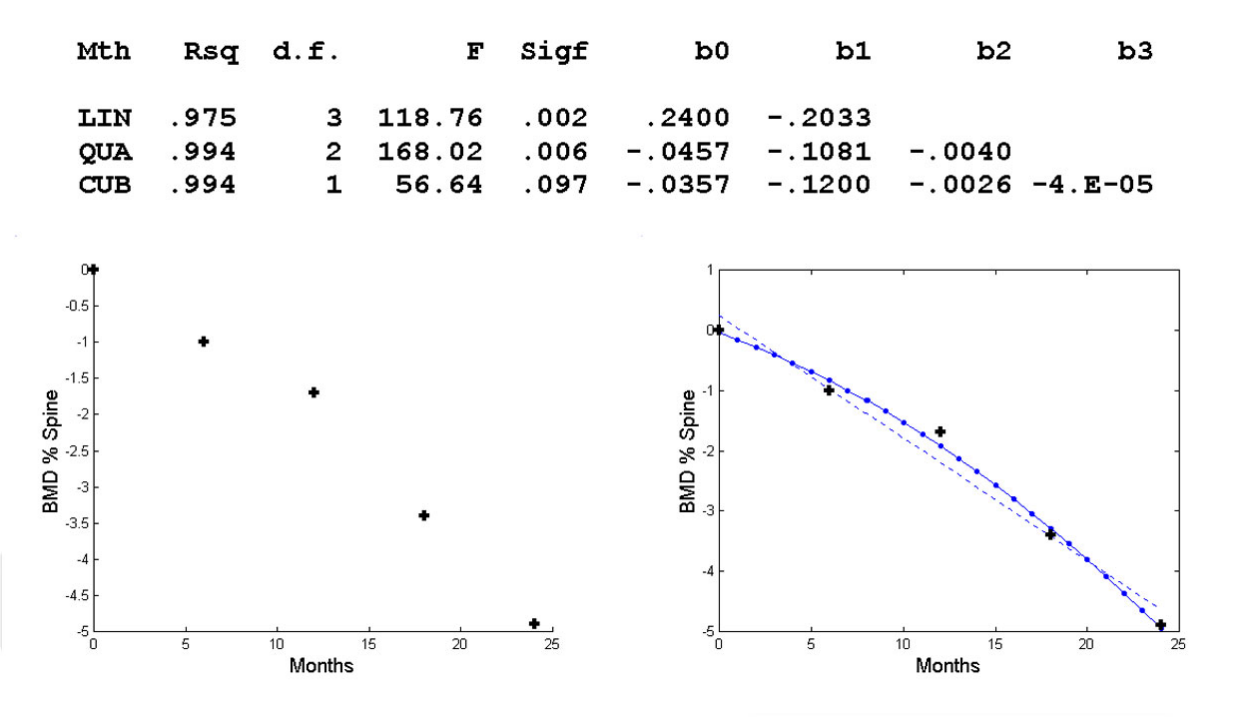
**Changes in BMD with respect to time in the spine**. Polynomial regression for changes in regional BMD with respect to time in the spine. The observed data (+) and the linear (--), quadratic (-.) model.

**Figure 5:**
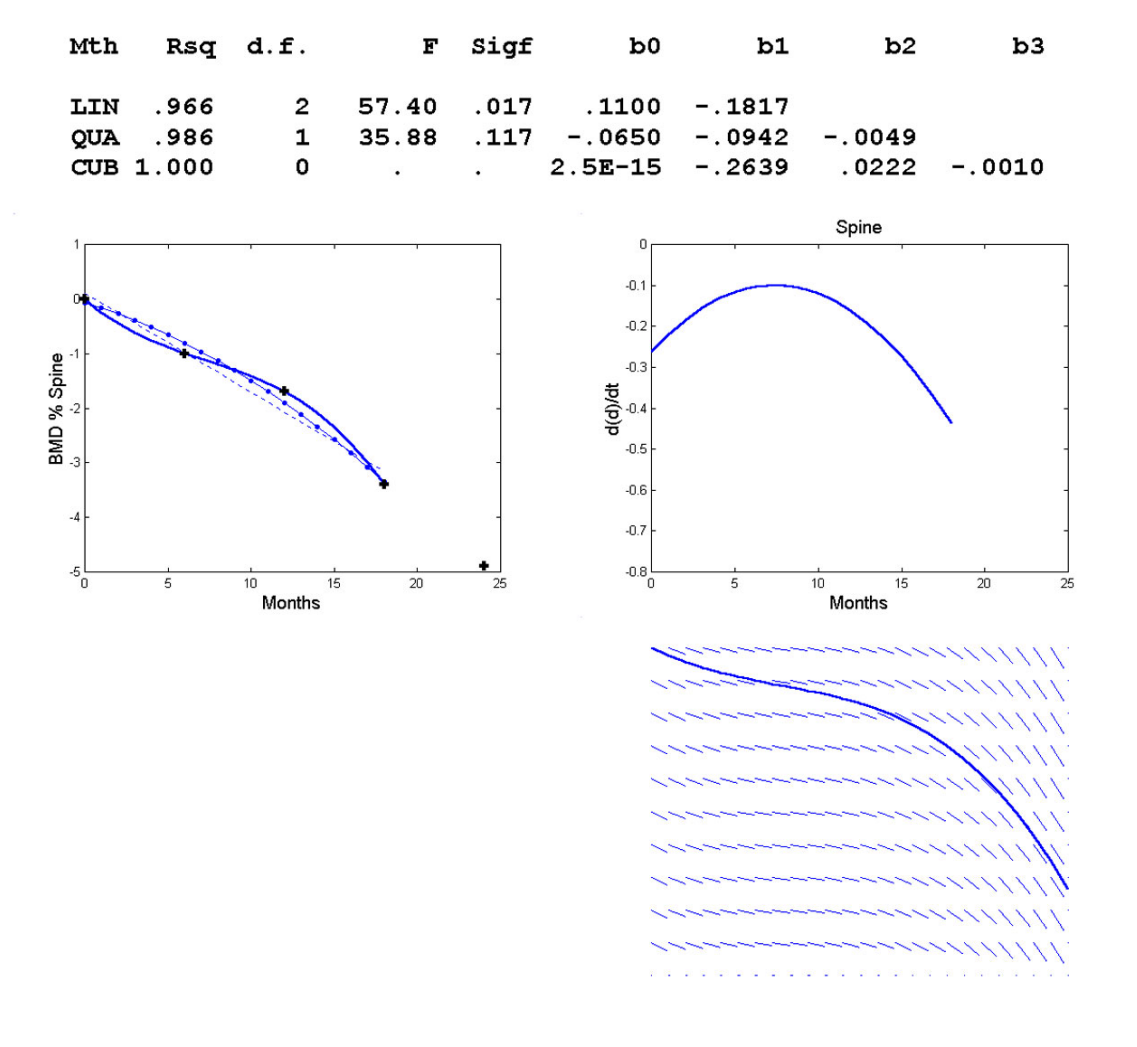
**Changes in BMD with respect to time in the spine with shorter time interval**. Polynomial regression for changes in regional BMD with respect to time in the spine. The observed data (+) and the linear (--), quadratic (-.) and cubic (-) model, graph of *f*'(*t*) vs. *t *and direction field for the spine. Time interval [0,18].

For the third model, BMD changes in the spine were estimated as a quadratic response (Figure 4). The equation of the curve was *d *= -.0457-.1081*t*-.004*t*^2^. Despite the high R^2^, it seems that the graphical model can be improved. Thus, we limited the time interval to [0,18] instead of [0,24] and derived the equation *d *= -.2639*t*+.0222*t*^2^-.0010*t*^3 ^(Figure 5). This cubic model provides a better graphical result and seems to be more compatible with the curve of BMD changes at the spine (Figure 1) until the 18^th ^month of therapy. There is a point of inflexion at t = 7.4 or about 7 months.

**Figure 6:**
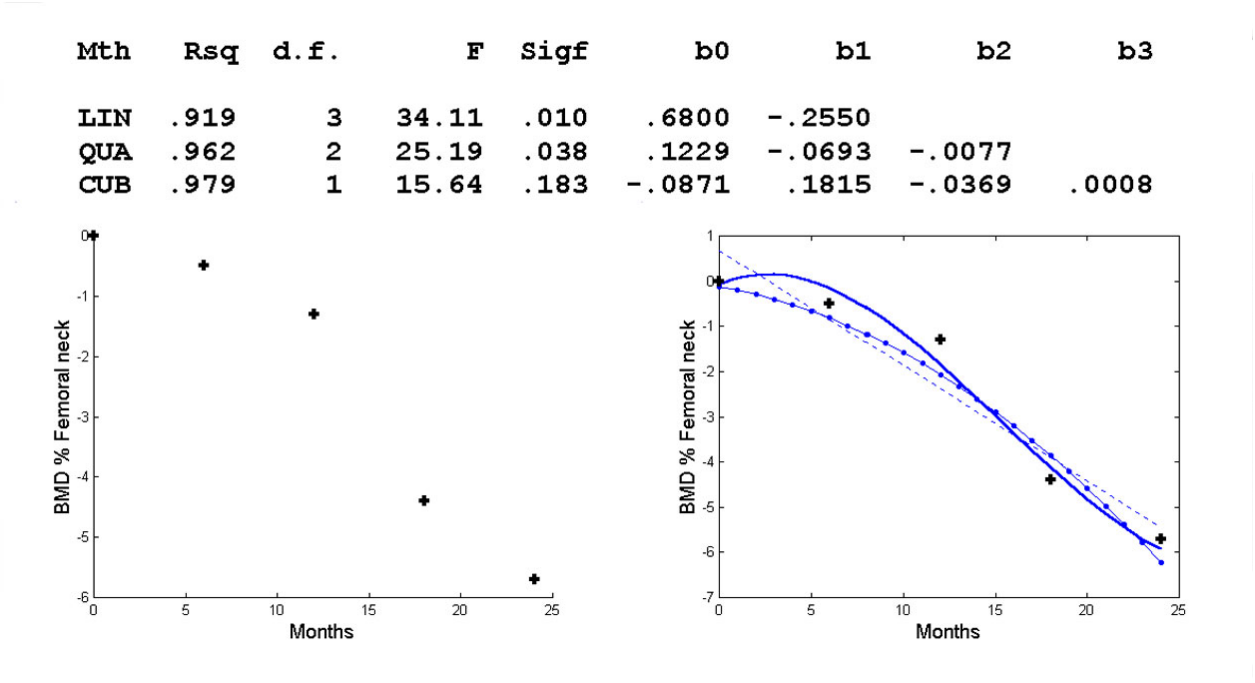
**Changes in BMD with respect to time in the femoral neck**. Polynomial regression for changes in regional BMD with respect to time in the femoral neck. The observed data (+) and the linear (--), quadratic (-.) and cubic (-) model.

**Figure 7:**
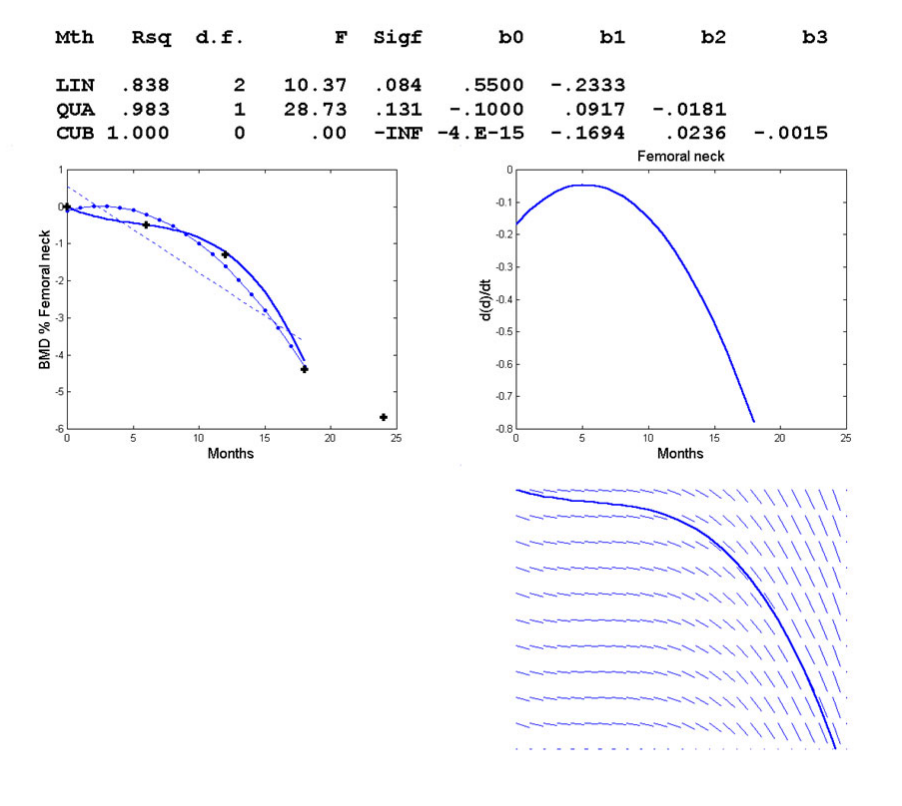
**Changes in BMD with respect to time in the femoral neck with shorter time interval**. Polynomial regression for changes in regional BMD with respect to time in the femoral neck. The observed data (+) and the linear (--), quadratic (-.) and cubic (-) model, graph of *f*'(*t*) vs. *t *and direction field for the femoral neck. Time interval [0,18].

For the femoral neck, the regression curve is *d *= -.0871+.1815*t*-.0369*t*^2^+.0008*t*_3 _(Figure 6). Again we got better results by choosing the time interval [0,18] (Figure 7). The regression equation is *d *= -.1694*t*+.0236*t*^2^-.0015*t*^3 ^and an inflexion occurs at *t *= 5.2444 or about 5 months.

## Discussion

Osteoporosis affects 75 million people around the western world and Japan, many of whom are unaware of the diagnosis until they suffer a life-altering fracture [31]. It quietly progresses without symptoms until late stage complications occur. The annual economic burden of osteoporosis in the United States alone exceeds that of congestive heart failure, asthma and breast cancer combined [32]. Therefore, the diagnosis, treatment and prevention of osteoporosis is a national health emergency. Bone mass density measurements have helped define a prefracture diagnosis of osteoporosis to predict fracture risk in postmenopausal women and elderly men, and to monitor the course of disease processes that negatively affect bone or therapeutic agents that can improve bone strength. The fracture risk increases when suppressive doses of levothyroxine are administered especially in postmenopausal women. Suppression of thyrotropin secretion is indicated in patients with thyroid cancer, especially those with differentiated thyroid carcinoma, because these tumors may be dependent on thyrotropin [33]. Thyroxine is also given in an attempt to decrease the size of the thyroid in patients with diffuse or nodular goiter and to prevent regrowth after surgery.

The U.S. Preventive Services Task Force addressed screening for osteoporosis in postmenopausal women in 2002 [30]. Because of the tendency toward thyroid hormone-induced cortical bone loss, Greenspan and Greenspan recommended testing bone mineral density at a cortical site if only one site can be tested at a particular center. However, they stated that because national reimbursement guidelines are by visit and technology rather than by number of sites assessed, many centers routinely measure bone mass of both the hip and the spine for the same cost. No studies have specifically addressed the appropriate timing of follow-up bone mineral density in this patient population with TSH suppression. Standard guidelines for the prevention of osteoporosis suggest that follow-up be done in 1 to 2 years [29].

Most studies have reported bone loss in estrogen-deprived post-menopausal women taking suppressive doses of L-thyroxine. The variation of BMD in relation to the variation of time in these patients defines functions that can be formulated in mathematical terms. We based our models entirely on real world data. Data were used to suggest the models and estimate the values of parameters appearing in them. We then manipulated the model using mathematical techniques, graphical representation and computer aided numerical computation. Changes in regional BMD versus time at the spine, femoral neck, trochanter, and Ward's triangle in post-menopausal patients receiving T4-suppressive therapy yielded four cubic polynomial models. There are four points of inflexion on the graphs of the equations at about 7, 5, 5 and 6 months for spine, femoral neck, trochanter, and Ward's triangle, respectively. In other words, a maximum speed of bone loss is reached around the 6^th ^month after the start of suppressive L-thyroxine therapy in post-menopausal women. Thereafter, activation of compensatory mechanisms does not seem implausible. Low bone mass is an important risk factor for fractures and early identification of those individuals at risk of reduction in BMD caused by L-thyroxine may facilitate early therapeutic intervention. So it seems reasonable to check BMD at the 6^th ^month. The potential effects of L-T_4 _on long-term BMD necessitate more longitudinal studies and data to determine whether densitometry or biochemical markers during the first year of treatment can predict the degree of reduction of BMD. Our models' predictions of the time of maximum rate of change in bone mass density correspond reasonably well to each other. Apart from graphical and statistical fitness, this can be considered as a means to verify the models.

Any natural system can be studied phenomenologically in order to reach a deeper knowledge of its behavior. Models are simplified representations of certain aspects of real world problems. They help to predict what will happen in the future, or to estimate the effect of various factors on the observed phenomenon. Modeling is an iterative process and models may be simplified and refined repeatedly to provide generality and precision. There are no precise rules or exclusive techniques in mathematical or statistical modeling. In our study we have tried to show that choosing a different view of the same experiment may lead to additional clinical inference. A bone density test provides a numerical value for bone loss. Measurements can be easily, accurately and reproducibly taken from an individual patient to establish a diagnosis of osteoporosis. Some techniques take only a few minutes to perform enable the areas of the skeleton most at risk of fracture to be assessed. The density value for an individual patient can be compared with a normal reference range. Modeling the effects of therapeutic modalities on bone mass density provides a rational basis for optimal dosing strategies. Also, such models can provide starting point from which to design experiments investigating the underlying pathophysiology of osteoporosis.

## Conclusion

This paper suggests that there may be a critical point on the curve of BMD versus time in postmenopausal women receiving suppressive LT4 therapy and shows how it can be found using mathematical techniques. Much more scientific information and research is needed to delineate the cause, and to confirm the clinical application of this phenomenon. But, such an approach can be used as a guide for future experimentation with larger sample sizes. We believe that the sophisticated application of mathematical techniques may give useful solutions to difficult and complex clinical problems.

## Competing interests

The author(s) declare that they have no competing interests.

## Authors' contributions

BM developed the mathematical model and wrote the initial draft. VH, SMT and BL identified the clinical data and coordinated general edits and preparation of the final manuscript. All authors read and approved the final manuscript.
